# Identification of promoter targets of enhancers by epigenetic knockdown using TAL DNA binding proteins

**DOI:** 10.1186/1756-8935-6-S1-O12

**Published:** 2013-03-18

**Authors:** Eric M Mendenhall, Kaylyn Williamson, Deepak Reyon, J Keith Joung, Bradley E Bernstein

**Affiliations:** 1 Broad Institute, Cambridge, Massachusetts, 02139 USA; 2 Molecular Pathology, Massachusetts General Hospital, Boston, Massachusetts, 02114 USA; 3 Harvard Medical School, Boston, Massachusetts, 02114, USA

## Background

A fundamental question in biology is how an organism's genome controls cellular differentiation and identity to produce numerous distinct cell types. At the epigenome level, this is partially influenced by cell type specific regulatory regions marked by accessible chromatin. Recent genome-wide maps of chromatin modifications and DNA associated proteins have identified over a million candidate regulatory elements in the human genome. These DNA sequence elements are proposed to drive the activation or repression of nearby genes, however new tools are needed to do a thorough and systematic functional analysis of these elements in an endogenous context.

## Materials and methods

To directly test distal elements in a loss of function manner, we have fused the chromatin modifying enzyme lysine specific demethylase 1 (LSD1, also known as KDM1A) to transcription activator-like (TAL) effector DNA binding proteins. The TAL:LSD1 fusions are designed to specifically bind target DNA sequences and alter the nearby chromatin state, specifically demethylation of histone H3 lysine 4 di and mono-methylation (H3K4me2/1), a mark of distal enhancers. We have designed TALs to target over 50 putative distal enhancers. These constructs were individually transfected into the erythroleukemic cell line K562, after which we examined changes in H3K4me2 and H3K27acetylation by ChIP, or examined gene expression changes by RNA-seq. Additionally we designed TALs fused to a 3X FLAG epitope to do ChIP-seq analysis and determine the specificity of TAL targeting.

## Results

We show using TAL:3XFLAG ChIP-seq that binding of TALs is virtually exclusive to their target sequence. We show the TAL:LSD1 fusions have the ability to specifically decrease H3K4me2, as well as H3K27acetyl, another mark of distal enhancers, at their intended target sites. We find roughly 50% of TALs significantly reduce the chromatin modifications at the target enhancer, but not at non-target control enhancers. For a subset of these TAL:LSD1 fusions, we used RNA-seq to identify a nearby gene that is downregulated upon loss of the chromatin state at the enhancer. These nearby target genes show a range of decreased expression, allowing us to relatively quantitate the contribution on transcriptional activation of the target enhancer.

## Conclusions

By utilizing TAL DNA binding proteins fused to chromatin modifying enzymes, we can precisely alter the endogenous chromatin state of a locus. We use this to direct enzymes that remove active chromatin marks. This is in effect, creating a method to do “epigenetic knockdowns” analogous to the commonly used method of genetic knockdowns. The removal of active chromatin marks disrupts the function of distal enhancers, thereby allowing us to identify the promoters they regulate, and quantify the enhancer’s contribution to its target gene’s expression.

